# Cases Medical and Chirurgical; with Physiological, Anatomical, and Practical Remarks

**Published:** 1781-07

**Authors:** 


V. Fr. Chr. Stöllers, D. A. D. &c. *Beobachtungen und Erfahrungen aus der inneren und aüfferen Heilkunft, mit phyfiologifchen, anatomifchen und practifchen Anmerkungen.* i. e. *Cafes medical and chirurgical; with phyfiological, anatomical, and practical remarks. By* Fr. Chr. Stöllers, M. D. 8vo. Gotha, 1780.

WE have here accounts of eight cafes that occurred to the author in his practice: The firft relates to a difeafe which he names *Tabes chyluritica*, five *marcor cum hectica expenditione chyli.* It occurred in a man forty years of age, who was troubled with an excretion, of what our author fuppofes to have been chyle, from the urinary paffages, accompanied with violent pain in the loins, frequent vomiting, coftivenefs, anxiety, and feverifh heat. The urine difcharged refembled thin milk, and after a few hours depofited a light white fediment, which, upon being fhaken, was again perfectly mifcible miſcible with the watery part. The author ſuſpected a ſtone in the kidnies to be the cauſe of this complaint, but this opinion, he tells us, was ill founded. Soap, opium, bitters, mucilaginous remedies and clyſters were tried repeatedly, but without affording any relief. At length, after a few purges of rhubarb, manna, and Epſom ſalt, he adminiſtered the Peruvian bark in ſubſtance, with an opiate at bed-time, by which means the patient recovered. In a note the author has collected what has been ſaid by different practical writers concerning this diſeaſe.

The ſecond paper contains a deſcription of a very conſiderable prolapſus of the vagina, which included ſeveral large calculi. After theſe were taken out the complaint was eaſily cured.

Next follows an account of a peculiar ſtony excreſcence from a dens molaris in a widow ſeventy years old, who had loſt all her other teeth. This ſubſtance was ſeparated by an accident without the tooth's being affected. Some time afterwards a ſimilar maſs made its appearance a ſecond time, and the tooth becoming looſe, they were extracted together. The firſt excreſcence weighed two drachms and fifty grains; the laſt, fifty-four grains. Placed in the nitrous acid it did not effervefce, but became very foft and affumed a brimftone-like colour.

The cafe fucceeding this is of a woman who was repeatedly troubled with a præternatural retention and adhefion of the placenta. In her fecond labour, after an hour and a half's fruitlefs exertion on the part of the midwife to deliver the placenta, our author was requefted to give his affiftance. He found it adhering firmly on the right fide near the orifice of the uterus, but was able to bring it away without much difficulty. When extracted he obferved in it a ftrong tendinous excrefcence. The fame thing happened to the patient in three fucceffive labours. At the clofe of the paper the author takes occafion to point out the dangerous confequences of leaving the feparation of the placenta always to nature.

In the fifth paper we have an account of a boy who, after violent exercife and drinking freely of beer and brandy, was attacked with fymptoms of mania. He bit at every thing he came near, rolling his eyes in a wild manner and with great quicknefs, without uttering a fyllable. Half a drachm of ipecacoanha forced into his ftomach excited vomiting, and brought up a great deal of bread and fome cucumber, after which the patient became quiet.

The ſixth is a caſe of ſpurious aneuriſm of the left arteria œſophagea attended with peculiar ſymptoms. The nature of the diſeaſe was not diſcovered till after death.—After this follows a caſe of palſy of the tongue and right arm and leg, accompanied with convulſions. Theſe ſymptoms followed a rheumatic fever and jaundice. A cure was effected by chalybeates and antiſpaſmodics. In the eighth and laſt paper the author relates his ſucceſs with the cicuta in a caſe of the tinea capitis that had reſiſted a variety of other remedies.

In an appendix to the work we meet with ſeveral miſcellaneous practical remarks. The author obſerves, that in caſes of inveterate rheumatiſm, and likewiſe in ſome venereal complaints, he has experienced excellent effects from an extract prepared from the freſh juice of the flowers and leaves of the napellus cæruleus or blue monkſhood. In rheumatic caſes, he gives from four to eight grains of it combined with the ſedative ſalt of Homberg; and in venereal affections he mixes it with mercury.

In this appendix we alſo find a new method of preparing a tincture of cantharides, by firſt pouring water upon the flies, and after ſuffering them to digeſt a few hours, adding ſpirit of wine.

The

The author advifes a previous diftillation of the latter with balfam of Peru. Two drachms of the powder of cantharides to four ounces of brandy, or two ounces of fpirit of wine, and the fame quantity of water, are, we are told, the beft proportions for this tincture either for internal or external ufe.

The author adds fome obfervations on the ufe of the fublimate, and the *vinum antimoniale*. He prepares the latter in a manner very different from Huxham, by pouring Spanifh wine upon any quantity of *vitrum antimoniale*, and filtring the mixture, after fhaking it repeatedly, and fuffering it to ftand for fome days. He extols the efficacy of this remedy in cafes of fmall-pox, worms, catarrhal fever, and hooping cough.

